# Experimental Infections of Pigs with Japanese Encephalitis Virus Genotype 4

**DOI:** 10.3390/microorganisms12112163

**Published:** 2024-10-26

**Authors:** Paul M. Hick, Deborah S. Finlaison, Kate Parrish, Xingnian Gu, Philip Hayton, Tiffany O’Connor, Andrew Read, Jing Zhang, Zoe B. Spiers, Pedro Pinczowski, Angel L. Ngo, Peter D. Kirkland

**Affiliations:** 1 Virology Laboratory, Elizabeth Macarthur Agriculture Institute, New South Wales Department of Primary Industries and Regional Development, Menangle, NSW 2568, Australia; deborah.finlaison@dpi.nsw.gov.au (D.S.F.); kate.parrish@dpi.nsw.gov.au (K.P.); xingnian.gu@dpi.nsw.gov.au (X.G.); phil.hayton@dpi.nsw.gov.au (P.H.); andrew.j.read@dpi.nsw.gov.au (A.R.); jing.zhang@dpi.nsw.gov.au (J.Z.); peter.kirkland@dpi.nsw.gov.au (P.D.K.); 2 Veterinary Pathology Services, Elizabeth Macarthur Agriculture Institute, Menangle, NSW 2568, Australia; zoe.spiers@dpi.nsw.gov.au (Z.B.S.); angel.ngo@dpi.nsw.gov.au (A.L.N.)

**Keywords:** Japanese encephalitis virus genotype 4, viremia, serology, infection model, swine, pathogenesis

## Abstract

The emergence of Japanese encephalitis virus (JEV) in eastern Australia in 2022 caused extensive reproductive disease in pigs and is a threat to public health. Groups of weaned piglets were experimentally infected with the Australian outbreak strain of JEV (genotype 4). All pigs challenged at 5 weeks of age were infected after an intradermal injection of 1 × 10^5.5^ (*n* = 4) or 1 × 10^4.5^ TCID_50_/pig (*n* = 5). Intranasal instillation was less effective at this age, infecting 3/4 pigs with the same higher dose and 1/5 with the lower dose. Intradermal injection using 1 × 10^5.0^ TCID_50_/pig also infected 9/9 pigs at 11 weeks of age. Infection in all cases was confirmed by qRT-PCR of blood samples, which identified a viremia peak at 3–4 days and detected JEV-specific antibodies as early as 5 days after the challenge. The detection of JEV in oral and nasal swabs and in saliva from chew ropes was less consistent. JEV was detected in the tonsils of 21/22 infected pigs and was isolated from the tonsils of 9/9 pigs sampled 19 days after the challenge at 11 weeks of age. The infected pigs showed no clinical signs other than pyrexia on Days 4–6. Histopathology consistent with JEV infection was evident in the nervous tissues of all but two pigs sampled 28 days after the challenge and was characterized by meningitis, encephalitis and gliosis throughout the brain. Serological studies showed extensive cross-reactivity between JEV and Murray Valley encephalitis virus using blocking ELISAs. However, the determination of limiting-dilution titres allowed for the identification of the infecting virus. This in vivo infection model will be useful in evaluating JEV vaccines and for comparative pathogenesis studies with other JEV genotypes.

## 1. Introduction

Japanese encephalitis virus (JEV) is a mosquito-borne (predominantly *Culex* spp.) flavivirus that can cause fatal encephalitis in humans, with approximately 68,000 cases of the disease occurring annually in China and Southeast Asia [1]. Pigs are an amplifying host for JEV and the infection can cause reproductive and neurological disease in this species [2,3]. Apart from two small transient incursions into remote Northern Australia [4], JEV was considered exotic to mainland Australia before a large disease outbreak in early 2022 across Queensland, New South Wales, Victoria and South Australia [5]. This was declared a ‘communicable disease incident of national significance’ in March 2022 until June 2023, with 42 cases of human disease including 7 fatalities associated with JEV genotype 4 (JEV G4) infection reported during this period [6].

The incursion of JEV G4 into Eastern Australia caused large-scale economic and welfare impacts, with the disease detected on more than 80 pig farms [7], prompting an emergency animal disease response [8]. The disease caused extensive production losses in NSW, with 60% of piggeries experiencing impacts on porcine foetuses and neonates during the summer of 2021/2022 [9]. Most of the losses in pigs were due to abortions and stillbirths, which were often associated with severe congenital abnormalities. Neurological disease was sometimes observed in piglets soon after birth. Additionally, JEV can cause orchitis in boars [10], resulting in reduced fertility due to poor semen quality or viability. Apart from the reproductive impacts on boars, JEV infection does not frequently result in clinical disease in healthy non-pregnant adult or growing pigs. However, subclinical infections can occur and affected pigs may act as a reservoir for virus amplification which has public health implications [11].

Previously considered exotic to Australia, it is now highly likely that JEV will become endemic. Disease outbreaks and public health concerns will continue, particularly in large pig populations where population turnover results in a large number of naïve individuals every year [12]. As a vector-borne flavivirus that is spread by mosquitoes, vaccines provide a practical option for disease control. Vaccines are used extensively in pigs in Asia (especially Japan, Korea, Taiwan and China) where the virus is endemic [13,14]. In Australia, there are major biosecurity impediments to vaccine importation, particularly attenuated live virus vaccines for use in animals. Additionally, all vaccines used in animals are based on genotype 3 whereas the virus that has entered Australia is a strain of genotype 4 [15]. While there is believed to be cross protection between vaccines produced against different genotypes, this protection may not be optimal [16]. Our understanding of the epidemiology and pathogenesis of JEV in pigs is predominantly informed by the dominant JEV genotypes 1 and 3, with limited data on JEV G4 which has only recently emerging as a pathogen of pigs [5].

The objective of this study was to establish a reliable method for infecting pigs with JEV G4 and to identify aspects of the virology that could be used as markers to assess the efficacy of vaccines against this genotype. Data were also generated to assist the evaluation of diagnostic tests and to guide approaches to ongoing surveillance for JEV transmission.

## 2. Materials and Methods

### 2.1. Source and Management of Pigs

Male, large white–landrace crossbred piglets were acquired at approximately 4 weeks of age. They had previously had ear tags applied for individual identification and routine preventative health measures (iron injection, coccidiostat and porcine circovirus 2 vaccination). All piglets were the progeny of 10 gilts that had given negative results in serological tests for JEV and related flaviviruses (Murray Valley encephalitis virus (MVEV) and West Nile virus (WNV)). Further, each piglet was considered suitable for the trial based on negative serology for JEV at the time of weaning (*n* = 63). A random selection of 27 piglets was recruited for this study, beginning October 2022 after 1 week of acclimation in a biosecure accommodation.

Piglets were housed in small groups in biological containment level 2 indoor animal housing with additional specific biosecurity measures for the containment of JEV and personal protection. The rooms were fitted with raised wire mesh flooring and rubber matting. Food and water were supplied ad libitum from multiple feeders and nipple drinkers in each room. A commercial formulated pellet diet was supplied (Grolean weaner pellets and pig grower pellets, Rivalea, Corowa Australia). The air temperature was controlled by central air-handling, which was initially set at 30 °C for 4-week-old pigs and reduced by 2 °C per week until 20 °C, where it was maintained for the remainder of the trial. A heat lamp was provided until 10 weeks of age.

Animal trials were conducted with approval from the Elizabeth Macarthur Agriculture Institute Animal Ethics Committee (Project ID 341) and Institutional Biosafety Committee (Approval M22/07).

### 2.2. Experiment Design

Two separate experiments were conducted using piglets challenged with JEV at 5 or 11 weeks of age. Piglets challenged at 5 weeks of age were divided into 4 treatment groups to compare different routes of infection and doses (Table 1). The groups were as follows: (i) intradermal injection of JEV with a higher dose (1 × 10 ^5.5^ TCID_50_/pig, *n* = 4) or (ii) lower dose (1 × 10 ^4.5^ TCID_50_/pig, *n* = 5), and (iii) intranasal instillation of the same high and (iv) low doses of JEV (*n* = 4 and 5 pigs, respectively). The pigs were housed in separate rooms according to the route of JEV challenge but were not separated according to the dose. For the second experiment, pigs were challenged at 11 weeks of age by intradermal injection of 1 × 10^5.0^ TCID_50_/pig (*n* = 9). The pigs that were not selected for use in the JEV challenge studies were housed in different rooms under the same conditions and served as controls without JEV infection challenge.

The outcomes of the JEV challenge were determined by veterinary observations of pigs at least twice a day to identify clinical signs including daily measurements of rectal temperature. Samples used for laboratory analysis to determine the outcome for JEV infection were EDTA-treated whole blood collected daily for 10 days after the challenge and serum samples were collected weekly for serology, including prior to the challenge. Additionally, nasal and oropharyngeal swabs were collected daily for 10 days after the challenge of the 5-week-old cohort using plain cotton tip swabs that were placed into viral transport medium (phosphate-buffered gelatine saline containing antibiotics (PBGS)) [17]. Saliva samples were collected from oral chew ropes at various times after JEV infection had been confirmed; the ropes were placed in the rooms for 15–20 min and processed according to the manufacturer’s instructions (Tego swine oral fluids kit, ITL Biomedical, Reston, VA, USA).

To compare the serological responses with those of natural JEV infection, serum samples were also collected from a group of 20 pigs. These were introduced at 14 weeks of age from a JEV-free farm to a farm where JEV transmission was occurring. These pigs were sampled on the day of arrival and 26 and 35 days later. These serum samples were tested in the JEV, MVEV and WNV blocking antibody ELISAs.

### 2.3. JEV Infection Challenge

The current study used the Australian reference Japanese encephalitis virus isolate O-883/NSW/22 which was characterized as genotype 4 (Genbank accession number: OP904182). This virus was isolated from the brain of an aborted foetus collected on 24 February 2022 from a farm located at Corowa, NSW. A 10% (*w*/*v*) suspension of the brain was mechanically homogenized in serum-free cell culture medium containing antibiotics (M199, MP Biomedicals, Santa Ana, CA, USA). The homogenate was then clarified by centrifugation at approximately 2000× *g* at 4 °C for 20 min and filtered through a 0.2 µm filter before adsorption at 3 dilutions onto a mosquito cell line (*Aedes albopictus*, clone C6/36), as described in Section 2.4.3.

Pigs were challenged with JEV by intradermal (i/d) injection or intranasal (i/n) instillation with 1 mL of diluted cell culture-derived JEV. The virus stock used for challenge (O908) was obtained by one additional passage of the isolate O883 in C6/36 cells incubated at 30 °C in serum-free medium and aliquots were stored at −80 °C. The cell culture supernatant was titrated in C6/36 cells with immunoperoxidase staining (described in Section 2.4.3) to determine the 50% tissue culture infective dose (TCID_50_). The dose for administration in a 1 mL volume was obtained by dilution in serum-free cell culture medium immediately prior to use. The intradermal injections were performed using a 25-gauge needle to deliver 0.25 mL in 4 locations (2 injection sites on each of the left and right lateral flanks). For intranasal administration, a 3 mL syringe was used to instil 0.5 mL into each nostril, after which, the snout was elevated for 60 s.

### 2.4. Laboratory Assays

#### 2.4.1. Real-Time Reverse Transcription PCR (qRT-PCR)

Nucleic acids were extracted from 25 µL of EDTA-treated blood or from 50 µL of viral transport medium containing swabs or oral fluids using the MagMAX™ Viral RNA Isolation Kit (ThermoFisher Scientific, Waltham, MA, USA) and a semi-automated magnetic bead-based nucleic acid extraction system (KF96, ThermoFisher).

Testing for JEV RNA was performed using the universal JEV assay described by Shao et al. [18] for the detection of a 63 bp target in the NS-1 gene. Briefly, 5 µL of extracted nucleic acid was added to 20 µL of AgPath One-Step RT-PCR mastermix (ThermoFisher) containing 300 nM of forward (5′-GCCACCCAGGAGGTCCTT-3′) and reverse primers (5′-CCCCAAAACCGCAGGAAT-3′) and 200 nM of a probe (5′-FAM-CAAGAGGTGGACGGCC-MGB-3′). The assay was run for 45 cycles on an ABI 7500 Real-Time PCR system (Applied Biosystems, Foster City, CA, USA) using the cycling conditions recommended for the mastermix. Fluorescent data (FAM and ROX) were analysed using the 7500 Software v2.3 with a fixed manual threshold (0.05). Positive and negative results were differentiated based on examination of the normalized FAM amplification curve. To indicate the relative quantity of JEV RNA, a cycle threshold value (Ct) was assigned to positive samples for which amplification was detected within 40 cycles. The result was classified as inconclusive for Ct values >40 and negative if there was no evidence of amplification at 45 cycles.

An in-house internal RNA control assay (XIPC) was added to the sample lysis buffer during nucleic acid extraction [19] and was detected in a duplex reaction with the JEV qRT-PCR to identify inhibition of the PCR reaction or reduced efficiency of the nucleic acid extraction. When inhibition was detected, a new nucleic acid preparation was extracted from 25 µL of the undiluted sample and from 50 µL of a 1/10 dilution of the sample.

#### 2.4.2. Blocking ELISAs for Antibodies to JEV and Other Flaviviruses

Antibodies to JEV were detected with an in-house blocking ELISA (bELISA), previously described for West Nile Virus [20], with the following modifications: the antigen used was inactivated purified recombinant Binjari virus virions which contained a JEV PrME (pre-membrane and envelope protein) gene insert based on JEV genotype 3 [21]. The monoclonal antibody MAb 989 [22,23] was used to detect this antigen and blocking by the test serum provided results that are expressed as the percentage inhibition (PI%) relative to the reactivity of the negative control sample. A positive result was determined as PI values > 60%, a negative result as a PI < 40%, and the other samples were considered inconclusive. A quantitative measure of the level of JEV antibodies in samples that were positive at a 1/10 dilution was determined by testing a 2-fold dilution series starting from 1/10 to identify the highest dilution that gave a PI > 40% in the bELISA. As a pilot study, oral fluid samples collected from ‘chew ropes’ were diluted 1/5 and tested in the JEV bELISA.

The pig sera were also tested for antibodies to the related Australian flaviviruses according to previously described bELISAs for Murray Valley encephalitis virus (MVEV) [24] and West Nile virus (WNV) [20].

#### 2.4.3. JEV Virus Isolation, Amplification and Titration

Virus isolation was attempted on selected qRT-PCR positive samples to detect infectious JEV. The mosquito cell line (*Aedes albopictus*, clone C6/36; ATCC number: CRL-1660) was grown to approximately 80% confluence in cell culture tubes (Greiner Bio-One, Kremsmünster, Austria) and the growth medium was removed and replaced with 2 mL of maintenance medium (M199 containing 1% foetal bovine serum and antibiotics). Samples consisting of a 10% (*w*/*v*) homogenate of tissue in cell culture medium or undiluted swab fluid or saliva obtained from chew ropes were filtered using a 0.2 µm filter. Subsequently, 200 µL of the sample was added to the culture tubes and the cultures were incubated at 30 °C. At the end of a 7-day incubation, passaging of the cultures was completed by scraping the cells into the medium and 100 µL of the culture medium and cell suspension were adsorbed onto an 80% confluent monolayer of BHK_21_ cells. Evidence of virus replication in either C6/36 cells or BHK_21_ cells was confirmed by a reduction in Ct values in the JEV qRT-PCR assay. The culture supernatant was collected when there was evidence of cytopathology involving more than 50% of the monolayer; the culture supernatant was clarified by centrifugation and the virus was stored in 1 mL aliquots at −80 °C.

The JEV preparations used for infecting the pigs were titrated by inoculating C6/36 cells in 96-well plates with a series of 10-fold dilutions of the virus stock in cell culture growth medium. As cytopathology was not usually observed in C6/36 cells, virus replication was detected by in situ immunoperoxidase staining [25] using mAb 989 to detect the JEV PrM/E protein. Briefly, cells were fixed for 10 min by adding 3% formaldehyde and 0.1% NP-40 (ThermoFisher) and blocked with 5% skim milk powder in phosphate-buffered saline (PBS). The mAb in 1% gelatine PBS was added for 90 min at 37 °C in a humid chamber; after washing, the mAb was detected using peroxidase-conjugated rabbit antimouse IgG (ThermoFisher).

### 2.5. Post-Mortem Examination and Histopathological Evaluation

A post-mortem examination was conducted on selected animals. Fresh and formalin-fixed tissue samples were collected, including samples of all major organs including the reproductive system and comprehensive sampling of the central nervous system (whole brain and cervical, thoracic and lumbar spinal cord). Fresh tissues were stored at −80 °C after the collection of tissue swabs to test for JEV RNA by qRT-PCR. Tissues were fixed in 10% neutral buffered formalin for histological processing and sectioning. Initially, 3 pigs with confirmed JEV infection were euthanized for sampling 10 days after the challenge at 5 weeks of age. For euthanasia, intramuscular sedation with azaperone (Stresnil, Elanco, Australia) was used, followed by intravenous administration of pentobarbitone sodium (Lethabarb, Virbac, Sydney, Australia). Two pigs were selected from each of the high dose groups and one pig from each of the low dose groups (Table 1). The remaining pigs that had been challenged at 5 weeks of age were euthanized on Day 28 and those challenged at 11 weeks were sampled on Day 19.

Histological sections were prepared according to standard procedures, stained with hematoxylin and eosin (HE), digitally scanned using an Olympus Slideview VS 200 scanner, and visualized using Olyvia 4.1 software (Olympus, Tokyo, Japan). Brain sections from all animals, including rostral, mid and caudal cerebral cortex, diencephalon, hippocampus, midbrain, brainstem, cerebellum and cervical, thoracic and lumbar spinal cord sections were examined. Lesions were described and a grading score (− (absent), + (mild), ++ (moderate), +++ (severe)) was given to each section, focusing on viral-mediated lesions (i.e., inflammatory infiltrate, gliosis and necrosis). All remaining organs, including the reproductive tissues, were also evaluated.

## 3. Results

### 3.1. Acquisition of Pigs and JEV Inoculum

On arrival at the EMAI, the piglets weighed 7.27 ± 1.77 kg (mean and standard deviation; range: 3.49–10.60 kg). All were considered healthy based on clinical examination and veterinary supervision during acclimation. All piglets were confirmed to be free of antibodies to JEV, MVEV and WNV at the start of the trial based on blood samples collected after arrival. Additionally, weekly samples from the pigs held for challenge at 11 weeks remained negative for antibodies to these flaviviruses prior to the challenge.

The titre of the prototype Australian isolate of JEV after passage in C6/36 cells (O908) was 6.1 × 10^6^ TCID_50_/mL. The titre of the diluted inoculums measured after being held under the same conditions during the inoculation of the pigs was slightly lower than the intended dose for pigs at 5 weeks (1 × 10^5.3^ TCID_50_/mL (higher dose) and 1 × 10^4.1^ TCID_50_/mL (lower dose)) and as intended after challenging the pigs at 11 weeks of age (1 × 10^5.0^ TCID_50_/mL).

### 3.2. Clinical Observations

**Figure 1:**
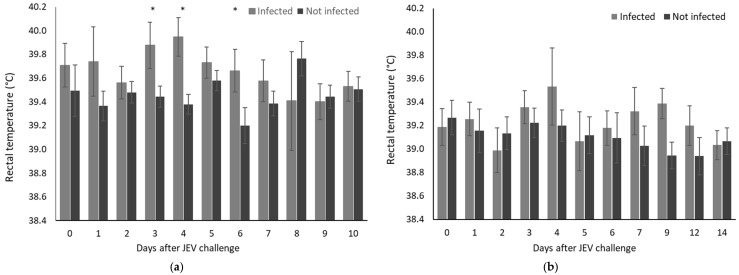
Rectal temperature of pigs measured daily after JEV challenge. Data are the mean and standard deviation; significant differences between infected and uninfected pigs on each day are indicated (*p* < 0.01) *. (**a**) Pigs challenged at 5 weeks of age were classified as infected or not infected based on the composite outcome of all laboratory analyses. There were 13 pigs that were infected by intradermal or intranasal challenge and 14 that were not infected (9 negative controls and 5 challenged by intranasal inoculation that showed no evidence of infection). (**b**) Pigs challenged at 11 weeks of age by intradermal injection were all infected (*n* = 9). Whilst there were no significant differences between infected and uninfected groups on any day, 3 of 9 infected pigs on Day 4 had pyrexia (temperature > 40.0 °C).

No signs of clinical disease were observed in any pigs challenged with JEV at 5 or 11 weeks of age. Pyrexia was detected in pigs infected with JEV between 3 and 6 days after the challenge at 5 weeks of age (Figure 1a). Compared to the control pigs, the mean temperature elevations were significantly higher on Days 3, 4 and 6. Pyrexia was also detected starting from 4 days after the challenge at 11 weeks of age in 3/9 infected pigs (≥40.2 °C), although overall, there were no significant differences between infected pigs in this age group and pigs that were not infected (Figure 1b).

### 3.3. Onset and Duration of Viraemia

JEV RNA was detected by qRT-PCR in EDTA-treated blood of all pigs after the intradermal challenge, commencing as early as 24 h after inoculation and continuing through to Day 7 in one animal (Table 2). The duration of viraemia in individual pigs ranged between 2 and 6 days. For pigs infected by the intranasal route, viraemia was detected between Days 4 and 9, with one exception. Virus isolation was not attempted on the positive blood samples (the Ct values were generally high: 33.1 ± 2.91).

### 3.4. Detection of JEV RNA in Ante-Mortem and Post-Mortem Samples

Nasal swabs were not effective for the detection of infection, although 6/13 pigs infected at 5 weeks returned at least one positive swab result during 10 days of daily sampling. Oral swabs were more reliable with at least 1 positive sample obtained from 12/13 pigs that were infected at 5 weeks. Saliva samples from chew ropes demonstrated that JEV RNA was present in the groups of challenged pigs after the viraemia had cleared and they had seroconverted (Table 3). Antibodies to JEV were also detected in these saliva samples. Virus isolation from the oral fluid samples that were positive for JEV RNA was not successful.

At the time of the post-mortem examination, JEV RNA was detected by qRT-PCR in the tonsils of all but one of the pigs that were shown to have been infected with JEV (Table 4). JEV was isolated from the tonsils of four pigs that were infected during the challenge at 5 weeks of age and from the tonsils of all the pigs challenged at 11 weeks of age, when sampled 19 days after the challenge. Each of these pigs had seroconverted 14 days earlier and included a sample with a low virus load (Ct = 34.6). There was limited detection of JEV by qRT-PCR in a broad range of other tissues collected at post-mortem, with tests of swabs of tissues rather than tissue homogenates having reduced sensitivity. There was a notable detection of JEV RNA in the prostate of one pig when sampled 19 days after the challenge at 11 weeks of age (Table 4).

### 3.5. Serological Response

The JEV specific serological response detected by bELISA had a rapid onset with antibodies present 5 days after the intradermal challenge in all 18 pigs (Table 5). The antibody response developed more slowly in four pigs that were infected by the intranasal route, which was detected in all the pigs that seroconverted by Day 9. The strength of the antibody response peaked 7 to 9 days after the challenge, with end-point titres as high as 10,240 which then declined to a relatively stable level through to the end of the study. Antibodies to JEV were also detected in oral fluid samples (Table 3).

The serial sampling from pigs up to 28 days after the challenge at 5 weeks of age provided 28 samples, of which, 25% were reactors in the MVEV bELISA (one was positive and six were inconclusive). In contrast, 58% (26/45) of all the serum samples collected to monitor the serological response of the pigs infected with JEV at 11 weeks of age were reactors in the MVEV bELISA (9 were positive and 17 were inconclusive). The PI values were consistently lower for the MVEV bELISA compared to the JEV assay, and the end-point titres when titrated were markedly lower in the MVEV bELISA compared to the JEV assay, which ranged from 10 to 320. No reactivity was detected in the WNV bELISA for any samples.

To further evaluate cross-reactivity in the JEV and MVEV bELISAs, the samples from pigs that were naturally exposed to JEV infection at 14 weeks old were tested (Table 6). All 20 were positive in the JEV bELISA when sampled 26 days from the potential time of exposure while 60% reacted in the MVEV bELISA (five were positive and seven were inconclusive). At Day 35, samples from the same pigs were again all positive in the JEV bELISA and 85% reacted in the MVEV bELISA (7 were positive and 10 were inconclusive), although the titres were higher for JEV (range: 320–2560) compared to MVEV (10–80).

### 3.6. Pathology

**Figure 2:**
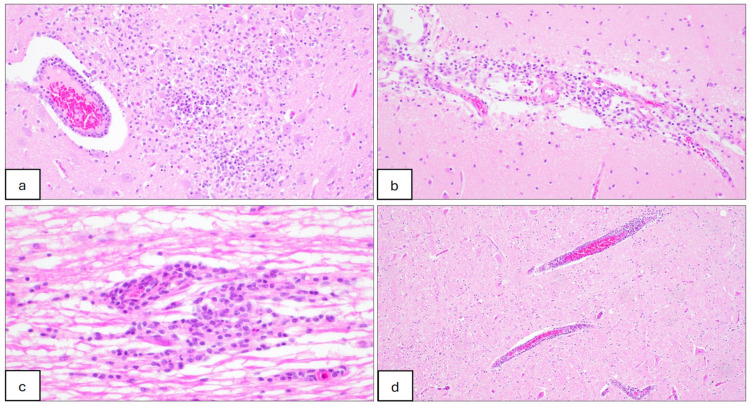
Histopathological lesions in the central nervous system of pigs infected with JEV at 5 weeks of age and sampled 10 days after challenge. (**a**) Brainstem section showing necrotising encephalitis. Focal extensive area of necrosis (malacia) adjacent to a lymphoplasmacytic perivascular cuffing, associated with moderate infiltration of macrophages, lymphocytes, plasma cells and a few neutrophils; HE, 20×. (**b**) Cerebrum section showing lymphoplasmacytic meningitis. Meninges are expanded due to moderate infiltration of macrophages, lymphocytes and plasma cells; HE, 20×. (**c**) Cervical spinal cord section showing lymphoplasmacytic myelitis. Mild to moderate focal infiltrate of lymphocytes, macrophages and plasma cells; HE, 40×. (**d**) Cerebrum section showing moderate lymphoplasmacytic encephalitis. Three vessels are cuffed by up to 3 layers of lymphocytes and plasma cells; HE, 10×.

**Figure 3:**
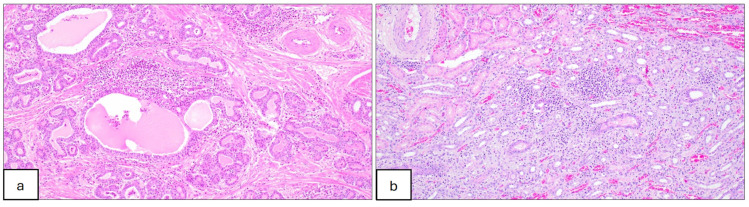
Histopathological lesions in tissues of pigs infected with JEV at 11 weeks of age and sampled 19 days after challenge. (**a**) Prostate section showing lymphoplasmacytic and neutrophilic prostatitis. Mild to moderate infiltrates of lymphocytes, plasma cells, macrophages and a few neutrophils surrounding prostatic glands. Glands are occasionally dilated and filled with eosinophilic fluid and a few necrotic cells. HE, 10×. (**b**) Kidney section showing lymphoplasmacytic interstitial nephritis. Mild to moderate infiltrates of lymphocytes, plasma cells and macrophages associated with mild fibrosis and surrounding tubules; HE, 10×.

No gross abnormalities associated with JEV infection were observed on post-mortem examination. Histological lesions suggestive of Japanese encephalitis were evident in the nervous tissues of all but 2 pigs that were confirmed to be infected, including 4/6 when sampled 28 days after challenge at 5 weeks of age and 9/9 that were sampled 19 days after the challenge at 11 weeks of age (Table 7). Lesions including lymphoplasmacytic perivascular cuffing (encephalitis), meningitis and gliosis were distributed predominantly throughout the brain and occasionally in the spinal cord for samples taken several days after the viraemia had cleared (Figure 2). Lesions suggestive of JEV infection were seen infrequently in other tissues; one pig challenged at 11 weeks had a lymphoplasmacytic interstitial nephrosis and another had lymphoplasmacytic and suppurative prostatitis (Figure 3).

## 4. Discussion

As a predominantly mosquito borne pathogen, the control of JEV infection is heavily reliant on the availability and application of effective vaccines. There is a need for detailed knowledge of the biology and pathogenesis of genotype 4 infection of pigs to identify key parameters that can be monitored to assess the efficacy of vaccines. There are advantages to using an experimental transmission model where the time, dose and route of infection are strictly controlled. At present, there are few studies of animals experimentally infected with JEV genotype 4 and these are limited to studies in mice [21,26]. In the present study, we showed that even using intradermal inoculation with a relatively low dose of cell culture-amplified JEV G4, young growing pigs can be readily infected. Intradermal injection most closely simulates infection by mosquitoes and results in a very high incidence of infection which allows small groups of animals to be used to achieve significant results. Additionally, an effective dose for infection by intranasal instillation of JEV provided another route that is more natural compared to some methods such as intravenous and intramuscular injections that bypass some components of the immune response. The rapid amplification of JEV that was detected as viraemia in a high proportion of young pigs highlights the role of pigs as an amplifying host in natural transmission cycles of JEV.

Consistent with experimental studies of pigs infected with other genotypes of JEV, the rapid onset and readily detectable viraemia which lasts only few days [27,28,29,30] provides a key parameter for identifying a productive infection. Additionally, the prolonged detection of virus in tonsils should provide a powerful measure of the efficacy of a vaccine in preventing the establishment of JEV infection.

The short duration of viraemia and rapid seroconversion followed by the development of high antibody titres limit opportunities to differentiate between historical and recent infection in pigs during diagnostic investigations. However, the persistence of virus in tonsils and its detection in oral swabs and saliva does provides a marker of recent infection and identifies a practical approach to JEV surveillance at a farm or at the population level. The use of chew ropes to detect viral nucleic acid and antibody in oral fluids has been adopted for surveillance purposes for many viruses [31]. The results of the present study confirm that this approach can also be used for the non-invasive detection of JEV RNA and perhaps antibodies. In this study, JEV RNA was detected in oral fluids beyond the period of viraemia and in some cases more than 3 weeks after infection. The detection of antibodies in oral fluids could be of value as a non-invasive method for surveillance in large pig populations to identify herds where there has been an active JEV infection or where there are large numbers of susceptible animals. However, more extensive studies are needed to establish the sensitivity of detection of both JEV RNA and antibodies in oral fluids as factors such as pen size, age, number of pigs, the number of ropes and sampling time all have an impact on sensitivity.

Although limited in extent and apparent efficiency compared to intradermal infection, the detection of infectious JEV in the oropharynx following inoculation by the nasal route support other studies [28,30] that highlighted a potential alternative transmission pathway directly between pigs, possibly involving superficial bite wounds. The presence of infectious virus in the oral cavity of pigs suggests that a low level of virus transmission could be maintained in the absence of insect vectors. Pigs also have a propensity to ingest reproductive material. Because there are potentially high quantities of JEV in foetal and placental tissues, ongoing transmission and higher infection rates might also occur in susceptible populations where animals are at critical stages of pregnancy.

Disease has generally been limited following postnatal experimental JEV infection of young pigs, ranging from a lack of clinical signs to moderate but transient illness. Despite these variable clinical effects, a consistent finding of experimental studies in pigs is mild to moderate histopathology changes in CNS tissues [28,29,30,32]. The lack of clinical signs with the detection of a range of histopathological changes in this experiment is consistent with these reports. During the Australian 2022 outbreak, while neurological signs were occasionally observed in newborn piglets, these manifestations were most likely an outcome of in utero infection. Disease in older, non-breeding pigs was not reported, raising the possibility that the JEV G4 outbreak strain (represented by O-0883/NSW/22) may be less virulent than some strains. This aligns with the current experiment, where most of the changes observed in the brain, though consistent with a viral infection, were mild. This interpretation is consistent with experimental studies in mouse models, where the NSW 2022 JEV G4 isolate was less virulent than isolates from genotypes 2 and 3 [21,26]. However, during the 2022 outbreak, there was still extensive and profound reproductive failure in pigs due to foetal infections and orchitis in boars and disease in horses and humans that included deaths. Further studies are recommended to evaluate the distribution and persistence of JEV using immunohistochemistry in key tissues including tonsils and male reproductive organs.

This study shows that definitive serological confirmation of active JEV infection remains challenging. Regardless of the age of pigs that were challenged, there was an extremely rapid seroconversion; initially, in the JEV bELISA, all pigs showed strong ELISA reactivity within 5 days of exposure. However, this was soon followed by low levels of reactivity in the assay for the related flavivirus MVEV. Although there was no evidence of prior or concurrent infection with MVEV, this cross-reactivity appears to increase with increasing age of the pigs. Within approximately 30 days of infection, samples from 85% of the 18-week-old pigs were reacting in the MVEV bELISA. However, the strength of cross-reactivity based on both the PI values and titres were markedly lower than those for JEV infections. This observation was reinforced by the surveillance undertaken during the outbreak where it was common to see almost all pigs in some populations had positive results in the MVEV assay. Without the concurrent use of assays for the detection of antibodies to both JEV and MVEV, there is potential for a failure to detect infection with either virus if relying solely on a qualitative result in a serological assay. While the percentage inhibition values consistently showed much stronger reactivity, and there was a higher proportion of reactors in the JEV bELISA, testing 2-fold dilutions of sera to establish ‘titres’ gave much more convincing differentiation between the serological response to the two viruses. In contrast, although also a closely related virus, cross-reactive antibodies to WNV were not detected in the assays employed. While a different genotype of JEV and a different monoclonal antibody were used for the JEV bELISA, collectively, the results of the present study are very similar to those described previously for pigs of similar age that were experimentally infected with JEV genotype 1 [33]. The known geographical distribution of MVEV is limited to Australia and Southeast Asia. This study demonstrated that antibodies that cross-react with MVEV will develop in pigs infected with JEV. This extensive cross-reactivity between viruses belonging to the JEV serogroup [34] make it important to be aware of the background serological status of animal populations for the presence of other flaviviruses that may confound the interpretation of serological results. Plaque reduction neutralization tests provide another approach to assessing the antibody response to JEV and related viruses and are important to predict vaccine efficacy [16]. The emergence of JEV in Australia has demonstrated the need for a One Health approach to diagnosis, surveillance and control of zoonotic disease [35].

## 5. Conclusions

In this study, we showed that JEV genotype 4 readily infects susceptible weaned and growing pigs when inoculated by the intradermal route, with a viraemia characterized by a rapid onset and a rapid antibody response that reached a high titre. Laboratory tests for viraemia and seroconversion were a reliable indicator of infection, together with histopathology suggestive of JEV infection. Together, this provides an experimental challenge model suitable for the evaluation of JEV vaccines in pigs. Further work is required to determine if the challenge model is suitable for pigs of all ages as the ultimate measure of an effective JEV vaccine for pigs is the prevention of foetal infections. This study also indicated the persistence of readily detectable levels of JEV RNA and infectious virus in the tonsils of infected animals many days after the clearance of the viraemia and seroconversion. This included the detection of virus components in saliva samples collected from chew ropes. This provides an additional method for disease surveillance, including the consideration of pig-to-pig transmission and over-wintering.

## Figures and Tables

**Table 1:** Experiment design and infection outcomes in pigs challenged with Japanese encephalitis virus genotype 4 at 5 or 11 weeks of age. The negative control pigs were not exposed to JEV and were housed under the same management conditions but in a different room. Specimens were collected from all pigs as indicated.

Age at Challenge	Challenge Method	Dose of JEV (TCID_50_/Pig)	No. Infected/No. Challenged	End-Trial Day (n)	Samples	Indicator of JEV Infection (n)		
						Viraemia	Tonsil ^a^	Seroconversion
5 weeks	Intradermal	1 × 10^5.5^	4/4	10 (2), 28 (2)	Oral and nasal swabs (Days 1–10) EDTA blood (Days 1–10) Serum (every 7 days) Oral fluid, tissues	4	4 (1/2)	4
		1 × 10^4.5^	5/5	10 (1), 28 (4)		5	4 (2/4)	5
	Nasal	1 × 10^5.5^	3/4	10 (2), 28 (2)		3	3 (1/1)	3
		1 × 10^4.5^	1/5	10 (1), 28 (4)		1	1	1
	None	No JEV	0/9	n/t	Serum (every 7 days)	n/t	n/t	n/t
11 weeks	Intradermal	1 × 10^5.0^	9/9	19 (9)	EDTA blood (Days 1–8, 10, 12, 14), serum (every 7 days) Oral fluid, tissues	9	9 (9)	9
	Control	-	0/9	23 (3)		0	0	0

^a^ qRT-PCR and virus isolation results for samples collected at the end of the trial. For 5-week challenge, VI was limited to samples collected at 28 days that were positive according to qRT-PCR; results are VI positive/tested by virus isolation. n/t: samples were not tested.

**Table 2:** Detection of JEV RNA in EDTA-treated blood samples collected daily after challenge by intradermal or intranasal administration. Positive qRT-PCR results are indicated by shading, data are the Ct values for positive samples. Neg: negative, JEV RNA not detected.

Challenge Method	Age at Challenge	Dose of JEV (TCID_50_/Pig)	Pig No.	Day 0	Day 1	Day 2	Day 3	Day 4	Day 5	Day 6	Day 7	Day 8	Day 9	Day 10
Intradermal	5 weeks	1 × 10^4.5^	1	Neg	Neg	34.2	35.6	34.4	35.7	Neg	Neg	Neg	Neg	Neg
			2	Neg	Neg	35.0	29.3	35.1	Neg	Neg	Neg	Neg	Neg	Neg
			3	Neg	36.9	32.1	29.0	37.3	Neg	36.3	Neg	Neg	Neg	Neg
			4	Neg	Neg	31.9	28.8	32.4	Neg	Neg	Neg	Neg	Neg	Neg
			5	Neg	Neg	29.9	29.0	Neg	Neg	Neg	Neg	Neg	Neg	Neg
		1 × 10^5.5^	6	Neg	34.4	30.0	28.6	30.9	37.2	Neg	Neg	Neg	Neg	Neg
			7	Neg	Neg	Neg	36.2	34.3	33.9	Neg	Neg	Neg	Neg	Neg
			8	Neg	Neg	32.0	30.2	33.4	Neg	Neg	Neg	Neg	Neg	Neg
			9	Neg	Neg	32.1	28.1	30.8	36.1	Neg	Neg	Neg	Neg	Neg
	11 weeks	1 × 10^5.0^	10	Neg	Neg	Neg	31.0	32.9	34.6	Neg	36.6	Neg	Neg	Neg
			11	Neg	Neg	Neg	36.0	32.9	37.0	Neg	Neg	Neg	Neg	Neg
			12	Neg	Neg	Neg	35.2	33.1	36.7	Neg	Neg	Neg	Neg	Neg
			13	Neg	Neg	Neg	33.7	35.0	Neg	Neg	Neg	Neg	Neg	Neg
			14	Neg	35.5	36.5	35.3	36.0	Neg	Neg	Neg	Neg	Neg	Neg
			15	Neg	Neg	Neg	Neg	36.8	33.2	Neg	Neg	Neg	Neg	Neg
			16	Neg	Neg	34.8	30.3	33.2	Neg	36.6	Neg	Neg	Neg	Neg
			17	Neg	Neg	Neg	33.8	34.0	35.5	Neg	Neg	Neg	Neg	Neg
			18	Neg	Neg	Neg	30.2	31.2	Neg	Neg	Neg	Neg	Neg	Neg
Intranasal	5 weeks	1 × 10^4.5^	19	Neg	Neg	Neg	Neg	36.4	33.4	33.8	Neg	Neg	Neg	Neg
			20	Neg	Neg	Neg	Neg	Neg	Neg	Neg	29.4	Neg	Neg	Neg
			21	Neg	Neg	Neg	Neg	Neg	Neg	36.3	Neg	Neg	Neg	Neg
			22	Neg	Neg	Neg	Neg	Neg	Neg	Neg	Neg	Neg	Neg	Neg
			23	Neg	Neg	Neg	Neg	Neg	Neg	Neg	Neg	Neg	Neg	Neg
		1 × 10^5.5^	24	Neg	Neg	Neg	Neg	Neg	31.3	28.0	Neg	36.2	Neg	Neg
			25	Neg	Neg	31.0	27.8	Neg	Neg	Neg	Neg	Neg	Neg	Neg
			26	Neg	Neg	Neg	Neg	Neg	31.3	29.0	33.25	Neg	34.7	Neg
			27	Neg	Neg	Neg	Neg	Neg	Neg	Neg	Neg	Neg	Neg	Neg

**Table 3:** Tests for JEV in oral fluids from ‘chew ropes’ from groups of pigs challenged at 5 or 11 weeks of age. Data are the cycle threshold (Ct) values for samples that tested positive for JEV RNA by qRT-PCR and the percentage inhibition (PI) in the JEV bELISA. n/t: not tested; Pos: positive; Neg: negative; Inc: inconclusive.

Age at Challenge	Group	Time (Days)	qRT-PCR Result (Ct)	Antibody ELISA	
				Result	(PI)
5 weeks	12 piglets, 7 infected	17	36.6	Pos	74
		18	Neg	Pos	84
		19	Neg	Pos	84
		20	35.9	Pos	84
		21	Neg	Pos	87
		24	37.2	Pos	92
		25	Neg	Pos	88
		26	Neg	Pos	87
		27	Neg	Pos	86
11 weeks	9 pigs, all infected	4	35.5	n/t	n/t
		5	32.5	n/t	n/t
		6	32.6	n/t	n/t
		7	36.2	n/t	n/t
		8	36.8	n/t	n/t
		9	33.7	Pos	66
		10	Neg	Pos	76
		11	35.0	n/t	n/t
		12	Neg	Pos	61
		14	Neg	Inc	52
		21	Neg	n/t	n/t

**Table 4:** Detection of JEV RNA by qRT-PCR in tissues collected from infected pigs at postmortem examination at various times after exposure to JEV. Data are the number of positive samples for each tissue type and the range for Ct values. Negative results were not tabulated and were obtained from all pigs from brain (cerebellum, cerebrum, diencephalon, olfactory bulb), spinal cord (thoracic, lumbar), sciatic nerve, testis, bulbourethral gland, vesicular gland and skin samples.

Challenge Method	Age at Challenge	Days After Challenge	No. of Infected Pigs Examined ^a^	Tonsil	Spleen	Thymus	Kidney	Ileum	Lymph Node		Spinal Cord—Cervical	Prostate
									Mesenteric	Sub-Mandibular		
Intradermal	5 weeks	10	3	3	0	0	1	0	1	0	0	0
				28.0–29.7	-	-	33.7	-	33.7	-	-	-
		28	6	5	0	1	0	1	1	3	1	0
				25.0–36.3	-	32.9		34.4	36.8	33.6–37.3	37.0	-
	11 weeks	19	9	9	0	0	0	0	n/t	n/t	2	1
				26.3–34.6	-	-	-	-	-	-	36.3–39.4	29.2
Intranasal	5 weeks	10	3	3	2	0	0	1	1	0	0	0
				28.3–32.3	33.6–35.7	-	-	36.5	33.3	-	-	-

^a^ JEV infection was defined as detection of viraemia (positive qRT-PCR) at any time from daily blood samples and seroconversion. n/t—not tested.

**Table 5:** JEV blocking ELISA results for determination of antibody titres in pigs at different times after challenge. The titre was determined as the highest 2-fold dilution of the sample that gave a result above the cut-off value (PI = 40%). Positive samples are indicated by shading with the titre shown. negative samples: Neg.

Challenge Method	Age at Challenge	Dose of JEV (TCID_50_/Pig)	Pig No.	Day 0	Day 3	Day 5	Day 6	Day 7	Day 8	Day 9	Day 10 ^a^	Day 14	Day 19/21	Day 28
Intradermal	5 weeks	1 × 10^4.5^	1	Neg	Neg	40	160	640	1280	1280	320a	640	640	640
			2	Neg	Neg	160	1280	5120	5120	2560	1280	1280	1280	1280
			3	Neg	Neg	320	1280	10,240	10,240	5120	2560	2560	5120	2560
			4	Neg	Neg	80	640	2560	5120	5120	1280	NS	NS	NS
			5	Neg	Neg	320	1280	10,240	5120	5120	5120	640	1280	2560
		1 × 10^5.5^	6	Neg	Neg	320	1280	5120	2560	2560	640	NS	NS	NS
			7	Neg	Neg	20	160	1280	2560	2560	640	640	640	1280
			8	Neg	Neg	160	1280	5120	5120	2560	1280	NS	NS	NS
			9	Neg	Neg	320	1280	10,240	5120	5120	1280	2560	2560	5120
	11 weeks	1 × 10^5.0^	10	Neg	Neg	160	640	2560	NS	2560	1280	640	1280	NS
			11	Neg	Neg	20	80	640	NS	2560	1280	320	1280	NS
			12	Neg	Neg	80	320	1280	NS	1280	640	320	640	NS
			13	Neg	Neg	80	320	1280	NS	1280	640	160	320	NS
			14	Neg	Neg	80	320	1280	NS	1280	1280	320	640	NS
			15	Neg	10	40	40	320	NS	1280	1280	640	320	NS
			16	Neg	Neg	160	640	10,240	NS	10,240	640	640	1280	NS
			17	Neg	Neg	80	320	1280	NS	1280	1280	320	640	NS
			18	Neg	Neg	21	80	640	NS	1280	640	320	640	NS
Intranasal	5 weeks	1 × 10^4.5^	19	Neg	Neg	Neg	Neg	10	80	640	2560	NS	NS	NS
			20	Neg	Neg	Neg	Neg	Neg	Neg	Neg	Neg	Neg	Neg	Neg
			21	Neg	Neg	Neg	Neg	Neg	Neg	Neg	Neg	Neg	Neg	Neg
			22	Neg	Neg	Neg	Neg	Neg	Neg	Neg	Neg	NS	Neg	Neg
			23	Neg	Neg	Neg	Neg	Neg	Neg	Neg	Neg	Neg	Neg	Neg
		1 × 10^5.5^	24	Neg	Neg	Neg	Neg	Neg	Neg	40	1280	NS	NS	NS
			25	Neg	Neg	Neg	160	2560	5120	5120	10,240	NS	NS	NS
			26	Neg	Neg	Neg	Neg	Neg	10	40	1280	5120	1280	5120
			27	Neg	Neg	Neg	Neg	Neg	Neg	Neg	Neg	Neg	Neg	Neg

^a^ Plasma from EDTA blood was used on Day 10; some pigs were euthanized on Day 10 and have no further samples (NS).

**Table 6:** Comparison of the serological response in JEV and MVEV blocking ELISAs for pigs that were naturally infected with JEV at 14 weeks of age. Data are the percentage inhibition (PI) at a starting dilution of 1/10 with darker shading for a positive result (PI > 60%) and lighter shading for an inconclusive result (PI 40–60%). The titre was determined for positive samples as the highest 2-fold dilution that gave a result above the cut-off value for a negative result (PI = 40%). All samples were also tested in the WNV blocking ELISA with negative results.

Pig No.	Day 0				Day 26				Day 35			
	JEV PI	JEV Titre	MVEV PI	MVEV Titre	JEV PI	JEV Titre	MVEV PI	MVEV Titre	JEV PI	JEV Titre	MVEV PI	MVEV Titre
1	24.8	<10	6.5	<10	93.2	1280	65.1	20	95.0	1280	74.0	40
2	−2.7	<10	−7.2	<10	84.3	640	36.5	<10	87.8	640	23.9	<10
3	29.6	<10	11.6	<10	87.9	1280	57.7	20	94.7	2560	49.0	10
4	−2.2	<10	−1.1	<10	92.9	1280	61.1	20	95.0	2560	43.5	10
5	3.3	<10	7.4	<10	92.8	640	39.3	<10	95.3	1280	56.3	10
6	−1.6	<10	24.4	<10	93.5	1280	23.8	<10	94.6	2560	41.8	10
7	1.2	<10	14.3	<10	86.6	640	29.5	<10	92.8	1280	15.8	<10
8	1.3	<10	7.3	<10	91.4	2560	84.1	80	94.4	2560	82.7	80
9	26.7	<10	−2.8	<10	92.5	320	30.0	<10	93.8	320	37.1	<10
10	21.2	<10	−0.2	<10	87.4	640	49.7	10	87.2	640	66.7	20
11	30.5	<10	−6.2	<10	88.3	320	38.6	<10	94.4	640	49.1	10
12	21.6	<10	−6.5	<10	90.7	1280	44.3	20	95.1	1280	40.3	10
13	16	<10	6.0	<10	93.9	640	55.7	10	86.8	1280	75.0	40
14	−1.6	<10	10.1	<10	92.8	1280	38.5	<10	95.0	1280	47.4	20
15	−1.1	<10	1.7	<10	93.2	2560	72.3	40	95.3	2560	66.8	40
16	−3.2	<10	−29.8	<10	93.6	2560	50.7	10	94.7	2560	60.3	40
17	−0.4	<10	−7.1	<10	93.2	1280	79.4	40	95.2	2560	83.7	40
18	22.3	<10	−7.6	<10	93.1	640	24.8	<10	95.2	1280	51.3	20
19	19.4	<10	−20.1	<10	90.6	640	57.9	20	90.6	640	53.0	10
20	28.1	<10	−7.0	<10	92.1	1280	45.7	10	95.1	1280	57.7	20

**Table 7:** Classification and severity of histopathological changes in the brain and spinal cord of the pigs that were confirmed to be experimentally infected with JEV. Data are (a) the severity of lesions for individual pigs and (b) the severity average according to different anatomical locations in the brain and spinal cord. Lesions were scored as −, absent; +, mild; ++, moderate; +++, severe.

(a)	**Challenge Method**	**Age at Challenge**	**Dose of JEV (TCID_50_/Pig)**	**Days after Challenge**	**No. of Infected Pigs Examined**	**Lymphoplasmacytic Meningitis**	**Lymphoplasmacytic Encephalitis**	**Gliosis**	**Malacia**
	Intradermal	5 weeks	1 × 10^4.5^	10	1	++	++	++	−
				28	4	+	−	−	−
						−	+	−	−
						−	+	+	−
						−	−	−	−
			1 × 10^5.5^	10	2	−	++	+	−
						−	+	++	−
				28	2	+	+	++	−
						−	−	−	−
		11 weeks	1 × 10^5.0^	19	1	+++	+	++	−
					2	++	+	+	−
					3	++	+	−	−
					4	++	+	+	−
					5	+	+	+	−
					6	+	++	++	−
					7	−	+	+	−
					8	−	++	++	−
					9	−	+	+	−
	Intranasal	5 weeks	1 × 10^4.5^	10	1	++	+++	+++	+++
			1 × 10^5.5^	10	2	+	+	+	−
						−	−	+	−
(b)	**Anatomical location**			**Challenge Age: 5 weeks**				**Challenge Age: 11 weeks**	
				10 days after challenge (*n* = 6)		28 days after challenge (*n* = 6)		19 days after challenge (*n* = 9)	
	Cerebral cortex		rostral	+++		−		++	
			mid	+++		+		+++	
			caudal	++		+		+	
	Diencephalon			++		+		+++	
	Hippocampus			++		+		+	
	Midbrain			++		−		++	
	Brainstem			+++		+		++	
	Cerebellum			++		+		−	
	Spinal cord		cervical	+++		−		++	
			thoracic	+		−		+	
			lumbar	−		−		++	

## Data Availability

The original contributions presented in the study are included in the article. Further inquiries can be directed to the corresponding author.

## References

[1] Campbell G.L., Hills S.L., Fischer M., Jacobson J.A., Hoke C.H., Hombach J.M., Marfin A.A., Solomon T., Tsai T.F., Tsu V.D. (2011). Estimated global incidence of Japanese encephalitis: A systematic review. Bull. World Health Organ..

[2] Park S.L., Huang Y.-J.S., Vanlandingham D.L. (2022). Re-examining the importance of pigs in the transmission of Japanese encephalitis virus. Pathogens.

[3] Williams D.T., Mackenzie J.S., Bingham J. (2019). Flaviviruses. Dis. Swine.

[4] Hanna J.N., Ritchie S.A., Hills S.L., van den Hurk A.F., Phillips D.A., Pyke A.T., Lee J.M., Johansen C.A., Mackenzie J.S. (1999). Japanese encephalitis in north Queensland, Australia, 1998. Med. J. Aust..

[5] Zhang W., Yin Q., Wang H., Liang G. (2023). The reemerging and outbreak of genotypes 4 and 5 of Japanese encephalitis virus. Front. Cell. Infect. Microbiol..

[6] Mackenzie J.S., Williams D.T., van den Hurk A.F., Smith D.W., Currie B.J. (2022). Japanese Encephalitis Virus: The Emergence of Genotype IV in Australia and Its Potential Endemicity. Viruses.

[7] Vallis R., Byrne N., Sanderson J. (2022). Australian Government, Department of Agriculture, Fisheries and Forestry, Emergency Animal Disease Bulletin No. 125. https://www.agriculture.gov.au/biosecurity-trade/pests-diseases-weeds/animal/ead-bulletin/ead-bulletin-no-125.

[8] Animal Health Australia (2020). Response Strategy: Japanese Encephalitis (Version 5.0). Australian Veterinary Emergency Plan (AUSVETPLAN).

[9] Cook H., Hayes D., Myer S., Weaver M., Wagstrom L. (2023). Potential Impacts of Introduction and Establishment of Japanese Encephalitis Virus in the United States Swine Herd. Swine Health Information Centre. https://www.swinehealth.org/wp-content/uploads/2024/03/2024-JEV-Economic-Assessment-SHIC-White-Paper-Final.pdf.

[10] Zheng B., Wang X., Liu Y., Li Y., Long S., Gu C., Ye J., Xie S., Cao S. (2019). Japanese Encephalitis Virus infection induces inflammation of swine testis through RIG-I-NF-ĸB signaling pathway. Vet. Microbiol..

[11] Mansfield K.L., Hernández-Triana L.M., Banyard A.C., Fooks A.R., Johnson N. (2017). Japanese encephalitis virus infection, diagnosis and control in domestic animals. Vet. Microbiol..

[12] Furlong M., Adamu A.M., Hoskins A., Russell T.L., Gummow B., Golchin M., Hickson R.I., Horwood P.F. (2023). Japanese Encephalitis Enzootic and Epidemic Risks across Australia. Viruses.

[13] Nah J.J., Yang D.K., Kim H.H., Song J.Y. (2015). The present and future of veterinary vaccines for Japanese encephalitis in Korea. Clin. Exp. Vaccine Res..

[14] Morita K., Nabeshima T., Buerano C.C. (2015). Japanese encephalitis. Rev. Sci. Tech..

[15] Yang D.K., Nah J.J., Kim H.H., Song J.Y. (2014). Inactivated genotype 1 Japanese encephalitis vaccine for swine. Clin. Exp. Vaccine Res..

[16] Fan Y.-C., Chen J.-M., Lin J.-W., Chen Y.-Y., Wu G.-H., Su K.-H., Chiou M.-T., Wu S.-R., Yin J.-H., Liao J.-W. (2018). Genotype I of Japanese Encephalitis Virus Virus-like Particles Elicit Sterilizing Immunity against Genotype I and III Viral Challenge in Swine. Sci. Rep..

[17] Kirkland P.D., Farrugia B., Frost M.J., Zhang C., Finlaison D.S. (2022). Multiplexed serotype-specific real-time polymerase chain reaction assays—A valuable tool to support large-scale surveillance for bluetongue virus infection. Transbound. Emerg. Dis..

[18] Shao N., Li F., Nie K., Fu S.H., Zhang W.J., He Y., Lei W.W., Wang Q.Y., Liang G.D., Cao Y.X. (2018). TaqMan Real-time RT-PCR Assay for Detecting and Differentiating Japanese Encephalitis Virus. Biomed. Environ. Sci..

[19] Gu X., Davis R.J., Walsh S.J., Melville L.F., Kirkland P.D. (2014). Longitudinal study of the detection of Bluetongue virus in bull semen and comparison of real-time polymerase chain reaction assays. J. Vet. Diagn. Investig..

[20] Read A., Finlaison D., Gu X., Hick P., Moloney B., Wright T., Kirkland P. (2019). Clinical and epidemiological features of West Nile virus equine encephalitis in New South Wales, Australia, 2011. Aust. Vet. J..

[21] Harrison J.J., Nguyen W., Morgan M.S., Tang B., Habarugira G., de Malmanche H., Freney M.E., Modhiran N., Watterson D., Cox A.L. (2024). An Australian genotype IV Japanese encephalitis virus chimeric vaccine protects mice against lethal challenge. NPJ Vaccines.

[22] Gould E.A., Buckley A., Higgs S., Sy G. (1990). Antigenicity of flaviviruses. Hemorrhagic Fever with Renal Syndrome, Tick- and Mosquito-Borne Viruses.

[23] Higgs S., Gould E.A. (1991). Differences in fusogenicity and mouse neurovirulence of Japanese encephalitis viruses. Arch. Virol..

[24] Hawkes R.A., Roehrig J.T., Boughton C.R., Naim H.M., Orwell R., Anderson-Stuart P. (1990). Defined epitope blocking with Murray Valley encephalitis virus and monoclonal antibodies: Laboratory and field studies. J. Med. Virol..

[25] Kirkland P.D., MacKintosh S.G. (2006). Australian and New Zealand Standard Diagnostic Procedure (ANZSDP). Ruminant Pestivirus Infections. https://www.agriculture.gov.au/agriculture-land/animal/health/laboratories/procedures/anzsdp/pestiviruses.

[26] Nguyen W., Gyawali N., Stewart R., Tang B., Cox A.L., Yan K., Larcher T., Bishop C.R., Wood N., Devine G.J. (2024). Characterisation of a Japanese Encephalitis virus genotype 4 isolate from the 2022 Australian outbreak. NPJ Viruses.

[27] Lyons A.C., Huang Y.-J.S., Park S.L., Ayers V.B., Hettenbach S.M., Higgs S., McVey D.S., Noronha L., Hsu W.-W., Vanlandingham D.L. (2018). Shedding of Japanese Encephalitis Virus in Oral Fluid of Infected Swine. Vector-Borne Zoonotic Dis..

[28] Ricklin M.E., García-Nicolás O., Brechbühl D., Python S., Zumkehr B., Nougairede A., Charrel R.N., Posthaus H., Oevermann A., Summerfield A. (2016). Vector-free transmission and persistence of Japanese encephalitis virus in pigs. Nat. Commun..

[29] Ricklin M.E., Garcìa-Nicolàs O., Brechbühl D., Python S., Zumkehr B., Posthaus H., Oevermann A., Summerfield A. (2016). Japanese encephalitis virus tropism in experimentally infected pigs. Vet. Res..

[30] Park S.L., Huang Y.-J.S., Lyons A.C., Ayers V.B., Hettenbach S.M., McVey D.S., Burton K.R., Higgs S., Vanlandingham D.L. (2018). North American domestic pigs are susceptible to experimental infection with Japanese encephalitis virus. Sci. Rep..

[31] Bjustrom-Kraft J., Christopher-Hennings J., Daly R. (2018). The use of oral fluid diagnostics in swine medicine. J. Swine Helath Prod..

[32] Yamada M., Nakamura K., Yoshii M., Kaku Y. (2004). Nonsuppurative Encephalitis in Piglets after Experimental Inoculation of Japanese Encephalitis Flavivirus Isolated from Pigs. Vet. Pathol..

[33] Williams D.T., Daniels P.W., Lunt R.A., Wang L.F., Newberry K.M., Mackenzie J.S. (2001). Experimental infections of pigs with Japanese encephalitis virus and closely related Australian flaviviruses. Am. J. Trop. Med. Hyg..

[34] Makino Y., Tadano M., Saito M., Maneekarn N., Sittisombut N., Sirisanthana V., Poneprasert B., Fukunaga T. (1994). Studies on serological cross-reaction in sequential flavivirus infections. Microbiol. Immunol..

[35] Pham D., Howard-Jones A.R., Hueston L., Jeoffreys N., Doggett S., Rockett R.J., Eden J.-S., Sintchenko V., C-A. Chen S., O’Sullivan M.V. (2022). Emergence of Japanese encephalitis in Australia: A diagnostic perspective. Pathology.

